# Magnetic Resonance Imaging in the Assessment and Management of Post-injection-Site Morphea

**DOI:** 10.7759/cureus.65423

**Published:** 2024-07-26

**Authors:** Praveen K Sharma, Sharmeela S, Evangeline P Christina, Dhivya Gunasekaran

**Affiliations:** 1 Radiology, Saveetha Medical College and Hospital, Saveetha Institute of Medical and Technical Sciences, Saveetha University, Chennai, IND

**Keywords:** post-injection site, disease staging, magnetic resonance imaging, localized scleroderma, morphea

## Abstract

Morphea, a form of localized scleroderma, can significantly affect individuals by causing skin tightening and discoloration. We describe the case of a 22-year-old woman who presented with progressive skin changes and discomfort in her right gluteal region following a history of an intramuscular injection in the right gluteal region. Clinical examination suggested morphea, prompting us to conduct an MRI to better understand the extent and nature of her condition. The MRI results revealed thickening of the skin layers and signs of inflammation, helping us differentiate between active inflammation and fibrosis. This case illustrates how MRI can provide crucial insights for managing morphea effectively.

## Introduction

Morphea is a rare inflammatory connective tissue disease that causes localized skin thickening and stiffness due to fibrosis. The number of new cases per million individuals varies between 4 and 27 each year. The progression of localized scleroderma (LS) comprises an initial inflammatory phase marked by hyperemia, succeeded by fibrosis, sclerosis, and, ultimately, atrophy [1,2]. Clinical presentations of LS vary widely and may be categorized as follows: circumscribed (deep and superficial), linear (containing scleroderma en coup de saber), generalized (four or more separate plaques greater than 3 cm), pan-sclerotic, and mixed forms. The etiology could be of varied types, of which the post-injection trigger is a rare environmental trigger factor [3,4].

Morphea can result in flexion contractures, pain, and severe disability because it affects the underlying structures. At the extreme end of the spectrum, the disease can proceed over years to produce severe functional, cosmetic, and psychological issues; growth retardation; permanent structural abnormalities; atrophy; and significant joint contractures [5].

## Case presentation

**Figure 1:**
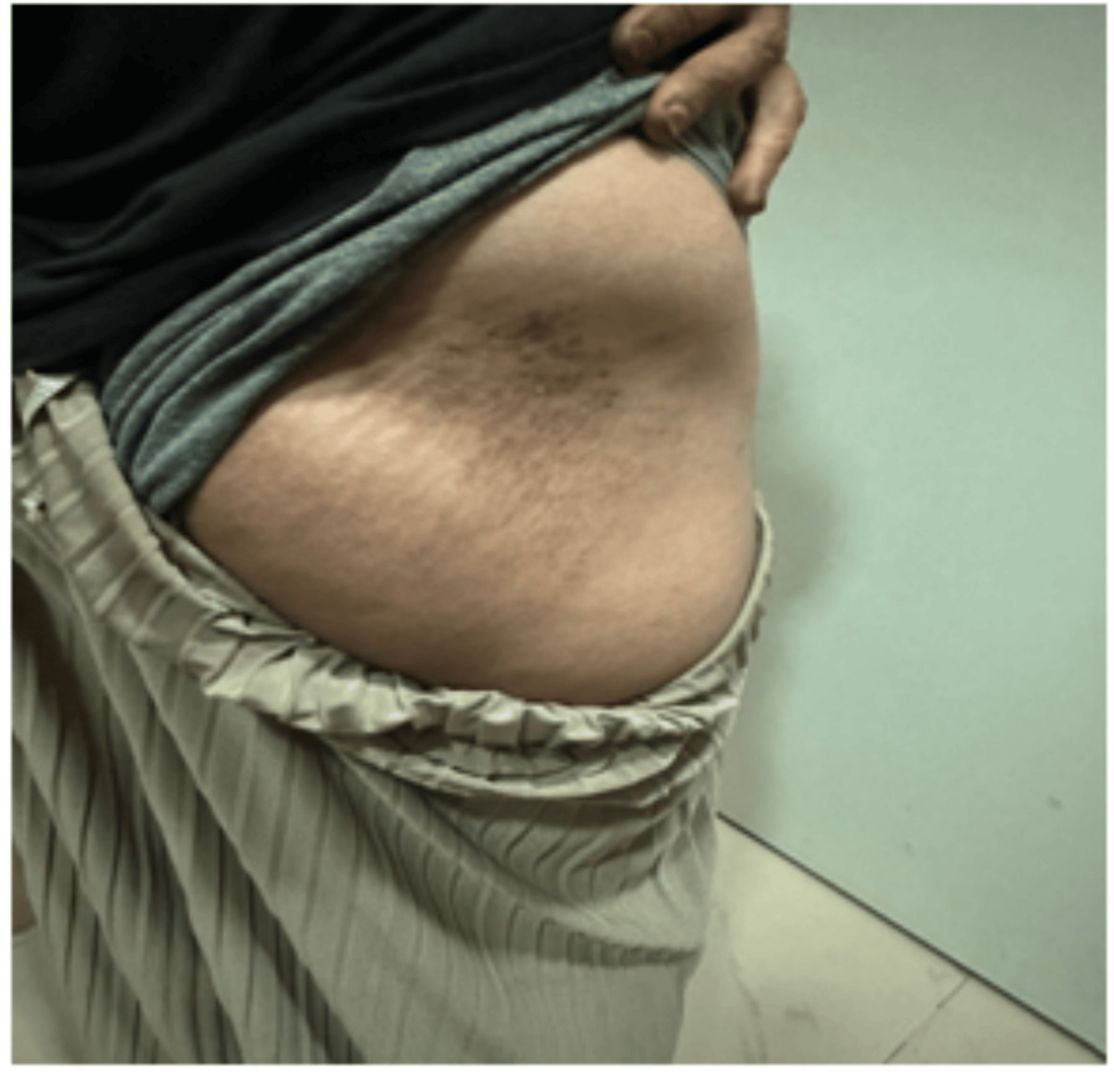
Focal skin discoloration with fat atrophy on the right lower back.

A 22-year-old young woman with localized scleroderma on treatment reported severe pain and color changes over her right buttock area. On examination, we noticed hyperpigmentation and local atrophy. She was on treatment with tablet mycophenolate mofetil 500 mg per oral twice daily for the past eight months. After a detailed history of the cause/environmental trigger factors, a history of intramuscular injection was elicited in the right gluteal region four years ago, followed by pain and clinical symptoms. A biopsy was done eight months ago and the histopathological examination revealed a thinned-out epidermis with orthokeratosis, with the dermis showing perivascular lymphoplasmacytic infiltrate predominantly in the lower dermis and the dermo-subcutaneous fat interface. Some of the blood vessels showed perivascular infiltrates by polymorphs and monophages. The collagen in the reticular dermis showed mild thickening and hypocellularity. Features suggestive of early lesions of morphea were reported. Laboratory investigations showed an elevated erythrocyte sedimentation rate of 45 mm/hour (normal = <20 mm/hour). Other parameters of complete blood count, renal function test, and liver function test were within normal limits. Given the need for staging/typing the morphea as superficial or deep to get a clearer picture of her present condition, we opted for an MRI using different imaging sequences. The superficial skin showed focal hyperpigmentation and atrophy in the right lower back (Figure 1).

**Figure 2:**
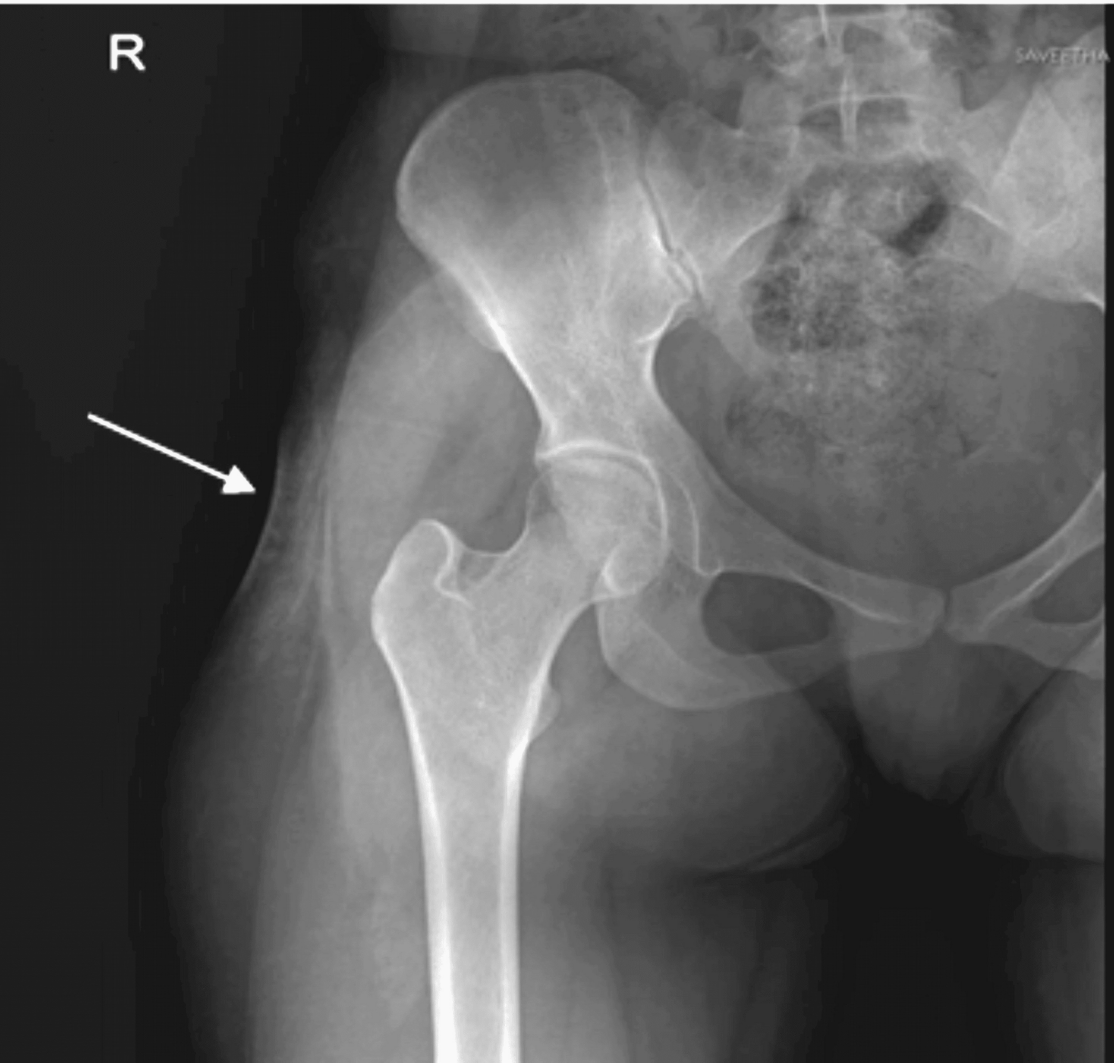
Radiograph of the right hip joint showing superficial soft tissue concavity in the lateral aspect with a focal increase in radiodensity.

A radiograph of the pelvis showed concavity in the lateral aspect of the right gluteal region (Figure 2).

**Figure 3:**
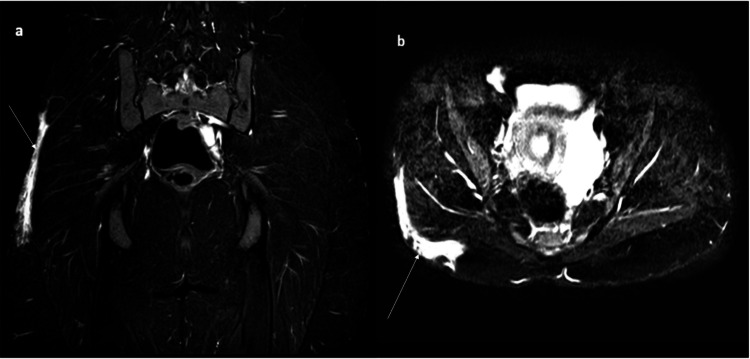
Short-tau inversion recovery (STIR) coronal (a) and axial (b) sections of the pelvis showing fairly defined areas of T2/STIR hyperintensity in the subcutaneous aspect of the right posterolateral aspect of the gluteal region (white arrow).

MRI showed T2/short-tau inversion recovery (STIR) hyperintensity in the subcutaneous plane and the overlying skin in the right lateral aspect of the pelvis (Figure 3).

**Figure 4:**
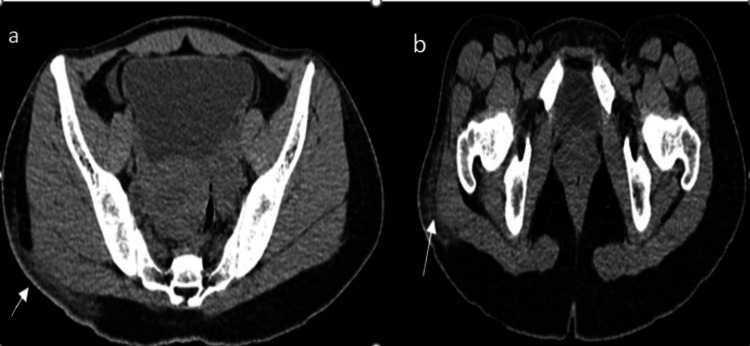
(a, b) CT showing a fairly defined area of fat stranding/inflammatory changes and relatively reduced subcutaneous fat in the posterolateral aspect of the right gluteal region.

CT screening of the patient showed fairly defined areas of fat stranding and inflammatory changes and relatively reduced subcutaneous fat in the posterolateral aspect of the right gluteal region (Figure 4).

The MRI revealed the involvement of the skin and subcutaneous tissues in the affected gluteal area, along with areas of increased signal intensity on certain MRI sequences (STIR) indicating inflammation. Specific sequences highlighted active inflammation in the superficial and deep layers extending along the iliotibial tract and lower back area. Additionally, some thinning of the fatty tissue beneath the skin suggested chronic changes. These MRI findings helped us distinguish between ongoing inflammation in this patient without any fibrosis, which is crucial for planning appropriate treatment with oral and topical immunomodulators. This patient was graded with an assessment score of 40% to 70% with moderate improvement. She has been advised to continue tablet mycophenolate mofetil and was prescribed topical tacrolimus 0.1% and ultraviolet B phototherapy.

## Discussion

Morphea is characterized by skin thickening and induration, with a complex pathophysiology involving genetic predisposition, immune dysregulation, vascular abnormalities, environmental triggers, oxidative stress, epidermal changes, and extracellular matrix remodeling. Genetic factors and abnormal immune responses play key roles in activating fibroblasts and promoting collagen overproduction, leading to fibrosis. Vascular damage and endothelial dysfunction contribute to tissue ischemia and perpetuate inflammation, while oxidative stress further exacerbates the disease process. Understanding these multifactorial interactions is crucial for developing targeted therapies, such as immunosuppressants, and emerging treatments, such as transforming growth factor-beta inhibitors, to address the underlying mechanisms of Morphea and improve patient outcomes.

Diagnosing morphea typically involves clinical examination and histopathological analysis of skin biopsies. Understanding the progressive nature of morphea is essential for effective management. The disease typically evolves through three stages. The first stage, inflammation, is marked by initial erythematous patches or plaques, signifying active inflammation. The second stage, sclerosis, involves skin thickening as well as induration due to the deposition of collagen. The final stage, atrophy, is characterized by advanced stages exhibiting shiny, thinned skin with visible blood vessels, where more profound involvement might result in bone, muscle, fat, and tissue atrophy [6].

Morphea presents in various forms, categorized primarily by the extent and morphology of skin involvement. The classification includes generalized, circumscribed, linear, and mixed types, each with distinct clinical presentations and prognostic implications. Circumscribed or limited plaque morphea, characterized by oval-shaped patches typically affecting two or fewer body sites, is the most typical variant seen in adults. Atrophoderma of Pasini and Pierini and guttate Morphea are examples of rare types that pose specific challenges for detection and treatment.

Accurate staging guides treatment decisions and predicts outcomes, highlighting the need for precise imaging modalities such as MRI to assess disease severity and progression [7,8]. MRI offers a non-invasive way to assess deeper tissue involvement and monitor disease activity. In this case, MRI provided clear images of skin thickening and inflammation patterns, supporting our clinical diagnosis and guiding our treatment approach.

Several diagnostic and evaluation tools, such as skin biopsy, elastography, ultrasonography, modified LS skin severity index (mLoSSI), and localized scleroderma cutaneous assessment tool (LoSCAT), have been recorded. These tools have limitations; for example, mLoSSI and LoSCAT evaluate only a small portion of the clinical parameters, and elastography is challenging to perform [9].

MRI findings helped us distinguish between ongoing inflammation and established fibrosis, which is crucial for planning appropriate treatment. Topical immunomodulators are opted for in treating superficial skin involvement and oral drugs in the involvement of deep muscular tissues [10,11]. There is no role of immunomodulators in the fibrosis stage of the disease. It has recently been demonstrated that MRI is a valuable assessment tool for determining the musculoskeletal involvement in patients with morphea [12]. MRI offers supplementary data on the extent to which underlying morphological structures are involved, in contrast to clinical examinations which often highlight superficial involvement characteristics. Therefore, MRI is often easier to understand and may validate findings from a physical examination.

Schanz et al. [13] highlighted that MRI offered additional information, which clinical examinations frequently fail to reveal, about the extent of involvement of morphological features. Thus, the ability of MRI to assess superficial or deep morphea and associated signal intensities based on grade and level of involvement makes it an essential tool.

## Conclusions

In this patient, MRI showed the prognosis of the treatment with immunomodulators and showed a clearer picture of superficial involvement of the skin and subcutaneous tissues without deeper extension. Further Follow-up MRI could assess the disease response to treatment and the natural course. The integration of MRI into clinical practice enhances the diagnostic accuracy of morphea and facilitates comprehensive disease management. MRI enabled clinicians to monitor treatment response by evaluating the efficacy of therapies such as corticosteroids and mycophenolate mofetil in reducing inflammation and preventing tissue fibrosis. It helped in predicting prognosis by assessing the likelihood of disease progression based on imaging findings, thereby guiding long-term monitoring and therapeutic adjustments. Longitudinal MRI assessments allow for early detection of disease recurrence or complications, prompting timely intervention to optimize patient outcomes.
